# Deficit of theory of mind after temporal lobe cerebral infarction

**DOI:** 10.1186/1744-9081-9-15

**Published:** 2013-04-22

**Authors:** Chunhua Xi, Youling Zhu, Chunyan Zhu, Daohui Song, Yongguang Wang, Kai Wang

**Affiliations:** 1 Neuropsychology Laboratory, Department of Neurology, The First Affiliated Hospital of Anhui Medical University, Jixi Road, Anhui Province, Hefei, 230022, China; 2 Department of Neurology, The Third Affiliated Hospital of Anhui Medical University, Huaihe Road 390, Anhui Province, Hefei, 230061, China; 3 Department of Psychology and Behavioral Sciences, ZheJiang University, Hangzhou, 310028, China

**Keywords:** Temporal lobe, Cognition, Theory of mind

## Abstract

**Background:**

Previous studies have indicated that the temporal lobe is involved in theory of mind (ToM). However, little attention has been paid to ToM in patients with cerebral infarction. In this study, we investigated the ability of ToM in patients with temporal lobe cerebral infarction (TLCI) using a variety of tests.

**Methods:**

In the study, 19 patients with TLCI and 20 healthy controls (HC) were examined using the Recognition of faux pas and the Reading the Mind in the Eyes (RME) tasks, to assess their ability of ToM.

**Results:**

The results of the study indicated that the TLCI group performed significantly worse compared with the HC group as revealed in the total faux pas-related score and in emotion recognition (Mind Reading).

**Conclusions:**

Our results implied that patients with TLCI had difficulty in ToM. Our data provided new evidence that the temporal lobe may be involved in processing ToM inferences.

## Background

Social cognition is defined as the process that modulates behaviour in response to conspecifics and, in particular, to the higher cognitive processes that subserve the extremely diverse and flexible social behaviours that are observed in primates [1]. A core component of social cognition is the capacity to attribute independent mental states to others or to predict other people’s behaviour based on their mental states, a capacity that is known as theory of mind (ToM) [2]. It can enable us to interact in complex social environments and to engage in the activities that we value most, such as family, friendship, love, and cooperation. Accordingly, impairments in ToM can have a serious negative effect on interpersonal relationships, employment, and social interactions [3,4].

Previous studies showed that traumatic brain injury [5] and various psychiatric disorders, including autism [6] and depression [7], are accompanied by deficits in ToM. Human lesion [5,8] and neuroimaging studies [1,9,10] have been pivotal in attempts to explore the neural substrate of aspects of ToM, such as the ability to recognize the emotional expressions and the reasons underlying the mental states of others. Evidence from several studies performed in both primates and humans suggested that the temporal lobe is involved in ToM and the recognition of emotion [11-14]. One of the first studies of higher-order ToM, which was reported by Schacher, found that patients with mesial temporal lobe epilepsy (MTLE) have impairments in their ability to recognize a faux pas, which is an advanced ToM task [15]. That study indicated that patients with MTLE, both pre- and postoperatively, performed significantly worse on the Faux pas test compared with patients with extramesiotemporal lobe epilepsy and healthy controls (HC), suggesting that MTLE is a specific aetiology of deficits in higher-order social cognition. Regarding the recognition of emotions, patients with anterior temporal lobectomy have deficits in emotion recognition [16]. Similarly, a few studies showed that patients with temporal lobe epilepsy had deficits in the ability to recognise emotions, ToM, and decision making [11,17-19]. These studies confirmed that widespread deficits in social cognition are common in MTLE. Moreover, a growing body of neuroimaging evidence has shown that the temporal pole was activated by a variety of stimuli: moral judgments, socio-emotional stories and sounds evoking a social scene [9,20,21]. Although many neuropsychological studies [11,18,19] examined ToM in individuals with MTLE, and functional neuroimaging studies [9,21] demonstrated that the temporal lobe was involved in ToM, fewer studies have explored the effects of temporal lobe cerebral infarction (TLCI) on ToM [22]. A study performed by Happe et al. [22] showed that people with right hemisphere (including the temporal lobe) stoke exhibited specific impairments in understanding stories and cartoons that require mental-state attribution. In addition, Weed et al. [23] reported on patients with right hemisphere stroke who had difficulty discriminating between film categories and a bias toward reduced mental-state ascription in the ToM condition. However, the location of the lesion in those patients was not limited to the right temporal lobe, and results from previous studies have not provided any conclusive evidence regarding the involvement of the temporal lobes in ToM.

Hence, based on the results of previous studies, the aim of the present study was to investigate ToM in patients with TLCI using a variety of tests, including the Recognition of faux pas and the Reading the Mind in the Eyes (RME) tasks. One Study from Pellicano et al. [24] showed that ToM development relied on executive function in children. Carlson et al. [25] implicated that inhibition control and working memory might be central to the relation between executive function and false belief understanding. Saxe and colleagues [26] have identified both separated and overlapping brain structures involved in ToM and executive function, suggesting that both domain general and domain specific cognitive resources are involved in ToM. In contrast, some lesion studies showed that ToM impairment was independent of executive function in adults [27,28]. Previous studies have shown that executive function might play an important role in ToM emergence during childhood, but mature theory of mind ability might not rely on executive functions [26]. However, a relationship between ToM and executive function or memory function has not been reported in patients with TLCI. Thus, we tested the relationship between other cognition functions and ToM in patients with TLCI.

## Methods

### Participants

The participants included 19 patients (3 females and 16 males) diagnosed with unilateral TLCI via head high-resolution magnetic resonance imaging (MRI). All patients were recruited from the Department of Neurology of the Third Affiliated Hospital of the Anhui Medical University. Exclusion criteria were current, or a history of, substance abuse, current or previous psychiatric diagnoses, colour blindness, intelligence quotient (IQ) as estimated using the Wechsler Adult Intelligence Scale-Revised Chinese Version (WAIS-RC) [29] below 80 (representing intellectual impairment), and history of diffuse brain damage. Sociodemographic-related information is summarized in Table 1. Among those 19 patients, 15 patients had structural brain damage within the right-side temporal lobe and 4 patients had left-side temporal lobe damage. Additionally, we recruited 20 healthy volunteers (7 females and 13 males, for the HC group) with similar cultural and demographic characteristics to those of the patients. They had no history of neurological problems, history of substance abuse, current or previous psychiatric diagnoses, colour blindness, or severe head injury. All subjects were right-handed and had normal or corrected-to-normal vision. The study was approved by the Anhui Medical University Ethics Committee, and participants gave written informed consent before the study and received financial compensation for participating in the experiment.

**Table 1:** **Demographic**, **neuropsychological tests for the TLCI and HCs groups** [**mean** (**SD**)]

	**TLCI**	**HCs**	**Statistics value**	***P *Value**
Age[years]	55.16 (14.04)	56(6.74)	F_(1,37)_=0.058	0.81
Gender (M,F)	16,3	13,7	—	0.35
Education[years]	10.11(3.33)	10.95(2.31)	F_(1,37)_=0.89	0.35
Time Since Lesion [days]	36.42(8.91)	—	—	—
IQ	97.84(8.03)	100.5(7.81)	F_(1,37)_=1.09	0.32
NIH Stroke Scale	4.68(2.94)	—	—	—
MMSE	29.0 (0.75)	29.25 (0.85)	F_(1,37)_=0.94	0.35
HAMD	2.95 (0.91)	2.5 (1.19)	F_(1,37)_=1.72	0.19
verbal fluency	15.21 (3.08)	17.45 (4.88)	F_(1,37)_=2.89	0.09
Go-No-Go task	2.0(0.58)	2.6(0.5)	F_(1,37)_=12.02	0.001
Forward digit span	5.68(1.16)	6.75 (1.29)	F_(1,37)_=7.33	0.01
Backward digit span	3.37 (1.07)	4.9 (1.68)	F_(1,37)_=11.39	0.002
AVLT				
Trial 5	8.95 (1.90)	11.05(2.19)	F_(1,37)_=10.22	0.003
Delay recall	8.32 (2.08)	9.7(2.38)	F_(1,37)_=3.71	0.06
Delay recognition	12.89(1.37)	13.65(1.63)	F_(1,37)_=2.44	0.13

### Background and neuropsychological testing

The following neuropsychological tests were administered to all subjects and compared between the HC group and the patients with TLCI: (1) full neurological examination, including the NIH Stroke Scale (NIHSS), was performed by a certified investigator to assess the neurological state of patients with TLCI [30]; (2) the Mini-Mental State Examination (MMSE) [31] was used to measure global cognitive functions; (3) the Hamilton Depression Scale [32] was used to measure depressive states; (4) verbal fluency (number of animals named/min) [33] was used to measure frontal functions; (5) the Go-No-Go task was used to estimate the ability of inhibitory control [34]; (6) the Digit Span test [29] was used to estimate short-term memory and executive function, including digital forward and digital backward; and (7) the Rey Auditory Verbal Learning Test (RAVLT) [35] was used to evaluate verbal memory.

### ToM tasks

*Recognition of faux pas task*: We used the previously published faux pas task [36] and adapted Stone’s Faux Pas task [37]. A faux pas occurs when a person unwittingly says something that should not have been said because it could hurt the listener’s feelings. An example of a social faux pas story is as follows:

‘*Lijing was at Wangfang*’*s home*. *While appreciating a crystal vase that she gave Wangfang as a birthday gift*, *she accidentally dropped the vase to the ground*, *which was then shattered into pieces*. *Lijing felt really sorry about breaking the vase*. *Wangfang said*, “*Don*’*t worry about it*. *I never like this vase anyway*.”’

In total, 10 faux pas stories were used in our study. The test administrator read the story aloud while the participant followed the story using his or her own print copy. After each story, the subject was asked a series of questions:

Question 1: did someone say something he (or she) should not have said?

Question 2: who said something he (or she) should not have said?

Question 3: why should he (or she) not have said it?

Question 4: why did he (or she) say it?

The last question (Question 5) was a control for story comprehension. Participants were asked a question about some important detail of the story without making inferences about the mental state of others.

After each story, participants were asked Question 1 (detecting the faux pas). If the participants answered yes, then they were asked Question 2 (identifying the correct person). If the participants identified the correct person, they were classified as having correctly identified the faux pas. Then, 2 follow-up questions were asked: Question 3 (testing that the participant understood that the listener would be hurt or insulted, which is an inference about the mental state of the listener) and Question 4 (testing that the participant understood that the faux pas was unintentional, which is an inference about the mental state of the speaker). If the subject answered No to Question 1, Questions 2–4 were skipped and Question 5 was immediately presented. In such cases, Questions 2–4 were assigned zero points. For scoring, we assigned 1 point for each correct response, producing scores for individual faux-pas–related scores (sum of the scores for questions 1–4 of all 10 stories). The scores on the 4 faux-pas–related questions indicated the general ability of participants to understand and represent the mental state of others. The faux-pas–related questions can be broken down further into 2 kinds of questions that determine whether or not the participant has correctly identified the faux pas and understood the faux pas [37]: Questions 1 and 2, which identified the faux pas (sum of the scores for questions 1 and 2 obtained for the 10 stories), and the scores for individual questions 3 and 4, which represented the understanding of the faux pas (sum of the scores for questions 3 and 4 of all 10 stories). All these scores were converted into a percentage of correct responses.

*RME task*: We used a previously published task for the evaluation of the expression of emotions in the eyes [7], i.e., an affective ToM task. A Chinese version of the Reading the Mind in Eyes task (RME) derived from Baron-Cohen [38]. This task comprises 34 photographs of Asians exhibiting various facial expressions, showing only the eye region (black-and-white photographs, 15 × 6 cm). For each eye region, participants were asked to choose from words that could describe the complicated emotion expression: 1 correct and 3 foil words. There were no time limits. At the same time, subjects were also asked to judge the gender of each person in each photo, as a control task. Before the formal test, we confirmed that the subjects were able to understand these words through vocabulary learning. The maximum score a subject could receive on the RME and gender recognition task was 34.

### Statistical analyses

Analyses were carried out using SPSS Version 11.5. Test scores are presented as means with standard errors. Effects were considered significant at p < 0.05. Normally distributed data were analysed using one-way analysis of variance (ANOVA). In the remaining cases, data were analysed using a non-parametric Mann–Whitney *U* test. Correlations between dependent variables were tested using Pearson’s correlation.

## Results

### Background and neuropsychological testing

**Figure 1:**
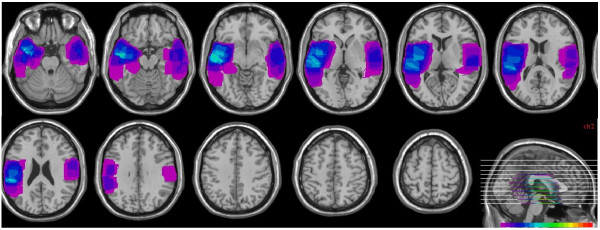
**Composite images of damaged brain regions in the group of patients with temporal lobe cerebral infarction.** Areas damaged in a subject are shown in purple; warmer shades denote the degree to which lesions involve the same structures in 2 or more individuals.

All patients had unilateral structural damage within the temporal lobe, as revealed by high-resolution MRI. Lesions were overlapped using MRIcrov.1.25 (http://www.mccauslandcenter.sc.edu/mricro/mricro/index.html) (see Figure 1). The lesion volume in the TLCI group was 32.66 ± 30.01 cc. The background and neuropsychological data of the TLCI and the HC groups are shown in Table 1. One-way ANOVA confirmed the absence of significant differences between the TLCI and the HC groups in terms of IQ, MMSE score, HAMD score, verbal fluency, and RAVLT delay recall and delay recognition (all p >0.05). As shown in Table 1, One-way ANOVA revealed significant group differences on measures of inhibitory control, digit span, and RAVLT trial 5 (all p < 0.05) (see Table 1).

### ToM tasks

**Figure 2:**
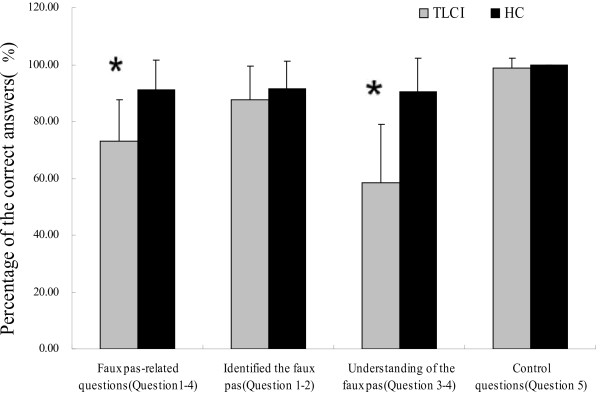
**Types of errors made on the faux pas task.** The TLCI group had impairment on faux-pas–related questions. The figure indicates that patients with TLCI could correctly identify the faux pas and answer the control questions. The TLCI group made more errors in understanding the mental states of the speakers and listeners than HC (Question 3 and 4) (*p < 0.001 vs. HC).

The results of these tests indicated that the TLCI group performed significantly worse on ToM compared with the HC group, as revealed in the total faux-pas–related scores (*U* = 59, p < 0.001). We found no significant differences between the TLCI and the HC groups regarding the control question (*U* = 170, p = 0.141). A separate analysis of the 2 kinds of faux pas questions—identifying the faux pas and understanding the faux pas—revealed other interesting findings. We observed no significant group differences in the questions aimed at identifying the faux pas (*U* = 146.5, p = 0.208). However, we found that there was a significant difference in the questions aimed at testing the understanding of the faux pas (*U* = 25, p < 0.001) (see Figure 2).

**Figure 3:**
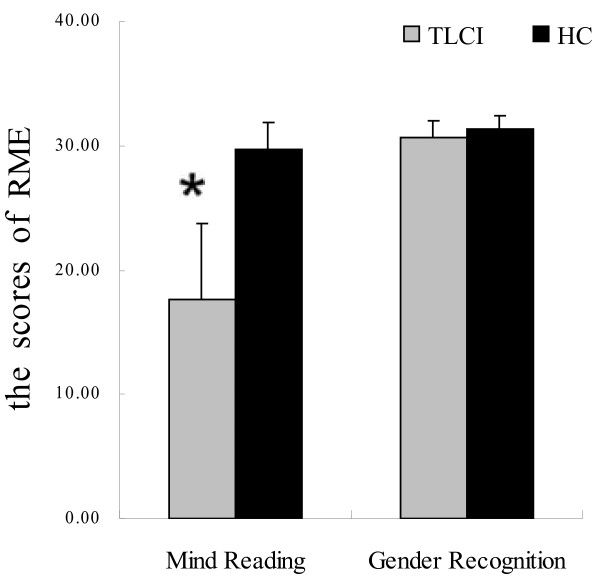
**Performance in the Reading the Mind in the Eyes (RME) test.** In the RME (emotion recognition) test, the performance of the TLCI group was worse than that of the HC group. However, there were no significant differences between the 2 groups in terms of performance in the gender-recognition task (*p < 0.001 vs. HC).

The RME task revealed the presence of a significant difference between the 2 groups, as the TLCI group performed worse than the HC group in emotion recognition (Mind Reading) (*U* = 0.00, p < 0.001). However, we found that there was no significant difference between the groups regarding gender recognition (*U* = 135.5, p = 0.114). These results suggest that patients with TLCI had difficulties representing the mental state of others based on eye expressions (see Figure 3).

### Correlation analysis

In this study, we also addressed the question of whether the deficit in ToM observed in patients with TLCI could be related to the neuropsychological test. Pearson’s correlation analyses were performed between tasks for this group. The only significant relationships found were those between the total faux-pas–related scores and the RAVLT trial 5 (*r* = 0.51, p = 0.02) (see Table 2). Since the patients not only had an impairment in faux-pas task but also in short term memory and inhibition performance, we used these factors as control factors for further partial correlation analysis. The results showed that there was still a correlation between the total faux-pas–related scores and the RAVLT trial 5 (Partial *r*=0.53, p = 0.02). However, we found that there was no significant relationship between the total faux-pas–related scores and the RAVLT trial 5 in the HC group(*r* = 0.16, p = 0.51). We found that there was no significant relationship between the faux-pas scores and the RME task. In addition, we found that there were no significant correlations between the ToM tasks and neuropsychological test in the control group (all p > 0.05) (see Table 3).

**Table 2:** Correlations between ToM and background and neuropsychological testing in patients with TLCI

									**RAVLT**				
	**Eyes mind**	**Eyes gender**	**Total faux pas-related**	**IQ**	**NIHSS**	**MMSE**	**HAMD**	**Verbal fluency**	**Trial 5**	**Delay recall**	**Delay recognition**	**Go-No-Go**	**Forward digit span**
Eyes mind	1												
Eyes gender	0.11	1.00											
Total faux pas-related	0.01	0.39	1.00										
IQ	0.14	0.01	−0.30	1.00									
NIHSS	0.05	−0.17	−0.22	0.08	1.00								
MMSE	0.29	0.17	−0.05	0.21	−0.28	1.00							
HAMD	0.16	0.30	0.21	−0.43	−0.21	0.16	1.00						
Verbal fluency	0.09	−0.10	0.33	0.30	−0.34	−0.15	−0.17	1.00					
Trial 5	0.21	0.38	0.51*	0.19	−0.04	0.43	0.06	−0.16	1.00				
Delay recall	0.17	0.22	0.32	0.14	−0.23	0.25	−0.20	−0.19	0.62**	1.00			
Delay recognition	0.16	−0.20	0.00	−0.08	−0.08	0.38	−0.13	0.18	−0.13	−0.14	1.00		
Go-No-Go	0.45	−0.07	0.00	0.08	0.13	0.00	−0.13	0.12	0.10	−0.09	0.07	1.00	
Forward digit span	0.35	0.08	−0.03	−0.06	−0.16	0.19	−0.12	0.16	0.17	−0.14	0.29	0.58**	1.00
Backward digit span	0.40	−0.11	0.00	−0.07	0.18	0.00	−0.26	0.14	0.26	−0.16	0.26	0.63**	0.59**

*Correlation is significant at the 0.05 level (2-tailed).

**Correlation is significant at the 0.01 level (2-tailed).

**Table 3:** Correlations between ToM and background and neuropsychological testing in the HC group

								**RAVLT**				
	**Eyes mind**	**Eyes gender**	**Total faux pas-related scores**	**IQ**	**MMSE**	**HAMD**	**Verbal fluency**	**Trial 5**	**Delay recall**	**Delay recognition**	**Go-No-Go**	**Forward digit span**
Eyes mind	1.00											
Eyes gender	0.04	1.00										
Total faux pas-related scores	0.06	0.37	1.00									
IQ	0.17	−0.02	0.13	1.00								
MMSE	0.04	0.30	0.38	0.14	1.00							
HAMD	0.37	−0.06	0.00	−0.09	0.49^*^	1.00						
Verbal fluency	0.08	−0.24	0.17	0.22	−0.12	−0.03	1.00					
Trial 5	0.12	0.17	0.16	0.16	−0.21	−0.05	0.14	1.00				
Delay recall	0.18	0.06	−0.09	0.14	−0.25	0.09	0.16	0.87^**^	1.00			
Delay recognition	−0.09	−0.16	0.20	0.12	0.03	0.23	0.25	0.43	0.36	1.00		
Go-No-Go	0.20	−0.12	0.08	0.29	−0.25	−0.26	0.16	0.45^*^	0.42	0.01	1.00	
Forward digit span	0.09	−0.12	0.06	0.15	0.30	0.26	0.17	0.23	0.18	0.66^**^	0.16	1.00
Backward digit span	0.22	−0.04	0.24	0.21	0.35	0.31	0.24	0.26	0.15	0.33	0.14	0.54*

*Correlation is significant at the 0.05 level (2-tailed).

**Correlation is significant at the 0.01 level (2-tailed).

## Discussion

This study examined the nature of mentalizing deficits presented by patients with TLCI, especially with medial temporal lobes lesions. Our results showed that the TLCI group had impaired ToM compared with the HC group. Consistent with previous literatures [11,13], our findings implied that the temporal lobe was involved in ToM.

The results from this study showed that the TLCI group had impaired performances on the total faux-pas–related test and the RME test compared with the HC group,while its performances on control question and gender recognition task were relatively normal. ToM ability can be further divided into 2 subcomponents: ‘cognitive ToM’ and ‘affective ToM’. Since the RME test is regarded as a classical task to measure affective ToM based on eye expression, the results of the current study indicate that affective ToM may be impaired in patients with temporal lobe infarction. In addition to this observation, our findings partially confirmed that the impaired performance on the faux pas test observed in the TLCI group was mostly related to a significantly worse performance on the question regarding understanding of the mental states of the speakers and listeners, and not to identifying the faux pas. Performing a social faux pas task may involve at least two levels of difficulty in cognitve loading processes: the identification and the understanding of a social faux pas. Identification of a social faux pas with questions 1 and 2 that merely ask whether the participants noted that there was a faux-pas at all and relied on the cognitive ToM ability. Understanding of a social faux pas maybe more requires, in addition, an empathic appreciation of the speaker’s and listener’s emotional state (knowledge about emotions). Following this line of thought, the poor performance on Questions 3 and 4 in patients with TLCI may be related to an increase of the ability to make affective representations of others’ mental states relative to Questions 1 and 2. The present study indicated that the TLCI group have a specific affective ToM deficit, which represented the deficit with the integration of emotional information for ToM. In social interactions, deficits in affective ToM may limit the TLCI patients’ abilities of understanding the intention of individuals to hurt the feelings of others. Subsequently, their social behaviours which are affected by misinterpretation of the intention to hurt possibly have an impact on the quality of social interactions and relationships [39].

Previous studies also showed the temporal lobe was involved in social emotional functions [40], such as in the perception of affective empathy of others [41], understanding of sarcasm [42], emotional expressions [43], and gaze direction [44,45]. A neuropsychological study also revealed that patients with atrophy in the temporal pole region [46] and patients with temporal lobe epilepsy [47,48] impaired in emotion recognition. One study performed in normal subjects using blood oxygen level-dependent functional MRI showed that areas including the temporoparietal junction, the superior temporal lobe, and the temporal pole were activated in affective empathy [14]. Our results may provide new evidence in support of the relationship between the temporal lobe and affective ToM.

Furthermore, the results obtained here also indicate that the deficit in the faux pas test observed in the TLCI group is similar to that of patients with dorsolateral prefrontal cortex lesions described in a previous study [5]. A functional neuroimaging study showed that the ToM task activated the right dorsolateral prefrontal cortex, the bilateral temporoparietal junction and the right inferior parietal lobule more than the non-ToM condition did [10]. Schilbach et al. [49] reported that not only the left medial basotemporal and right superior temporal cortex, but also the prefrontal lobe, were activated when participants viewed socially relevant clips. However, the deficit in the faux pas test observed in the TLCI group was different from that of patients with orbitofrontal cortex damage [8]. Patients with orbitofrontal cortex damage made errors in detecting faux pas by giving the answer that nothing awkward was said in the story [8]. Of course, the prefrontal cortex, together with areas of the temporal cortex, has been established as a part of the complex network underlying ToM, i.e., the ability to understand that others have beliefs, desires, and intentions that are different from one’s own [50,51]. In the present study the results indicated that the TLCI group could identify the occurrence of a faux pas in the story,but they could not correctly make inferences about the mental state of others. Therefore, it is conceivable that the deficits in ToM observed in the TLCI group were due to a disturbance in the connections of frontotemporal networks. Temporal lobe stroke may have an effect on higher-order social behaviour by altering afferents to frontal regions.

In addition, this study showed that patients with TLCI exhibited impairment not only in ToM, but also in executive-function subcomponents and memory. Within the TLCI group, the total faux-pas–related scores were correlated with RAVLT trial 5, indicating that memory impairment was associated with ToM. We did not find any correlation between the RME test or the faux pas test scores and other cognitive tasks. The results of this study indicated that the deficit of faux pas with the TLCI group might be related to auditory verbal memory impairment. Moreover, a neuroimaging study confirmed the existence of areas of activity that were common to autobiographical episodic memory and ToM within the frontal and posterior/medial temporal lobe regions [52]. However, we did not find that the correlation between the RME task and RAVLT in the patient group. The social faux pas task features verbal material and it requires participants to hold a large amount of verbal material in working memory in recognition of faux pas, while the RME task is thought to reflect a visual affective subcomponent of ToM [53]. The results from this study indicated that performance on the RAVLT 5 correlates with the faux pas task might be due to the verbal material of this task. We found that there was no correlation between ToM and neuropsychological testing including executive-function subcomponents and memory in the HC group. Previous studies have demonstrated development of ToM depends largely on the normal functioning of executive function memory systems in childhoods [54]. And some evidences have emerged to show that the ability of ToM seemed to be independent of a more cognitive or executive in normal adults [27,55]. The results from the study indicated that the ToM ability of the normal adults maybe not relied on executive or memory function. Moreover, the study also indicated that the RME test not correlated with the scores from the faux pas. Shamay-Tsoory et al. [56] have identified that the performance in affective ToM tasks was positively related to their empathic ability, indicating that the ability to make affective representations of others’ mental states is associated with the ability to empathize. The faux pas recognition test is thought to reflect both the cognitive and affective subcomponents of ToM. A rational explanation for this would be that performance of the RME test requires less empathic processing than is needed for the appreciation of a faux pas.

There are some limitations of our study. First, we could not use executive-function subcomponents and memory as covariates for analyses of the differences in ToM performance between groups because the data of the faux pas or RME was not normally distributed. Another limitation of the present study was that patients with unilateral lesions were not tested for hemispheric specialization for ToM because of the small sample of patient with left temporal lobe cerebral infarction in our study, although some researchers pointed out the role of right temporal lobe structures in social cognition [44,57] and impaired emotion processing and ToM ability in patients with right hemispheric stroke [23,58]. Moreover, previous studies indicated the presence of gender differences in social cognition [59]. Unfortunately, the sample size of the female patients with TLCI was too small to reach any conclusions of this nature.

## Conclusions

In conclusion, the results of the present study imply that patients with TLCI had difficulty in ToM, special in affective ToM. Our data contribute to new evidence that the temporal lobe may be involved in processing ToM inferences. Additional studies relying on both lesions and functional neuroimaging are required to elucidate these questions further in humans.

## Abbreviations

ToM: theory of mind; TLCI: temporal lobe cerebral infarction; HC: healthy controls; RME: Reading the Mind in the Eyes; RAVLT: the Rey Auditory Verbal Learning Test; MTLE: mesial temporal lobe epilepsy.

## Competing interests

None of the authors have any competing financial interests.

## Authors’ contributions

CZ, YW, and KW designed the experiments. CX YZ DS and KW conducted the experiments. CX YZ and DS analyzed the data. CX, YW, and KW drafted the manuscript. All authors read and approved the final manuscript.
